# Effects of an Iso‐Osmotic Chloride‐Free Solution With High Strong Ion Difference vs. Ringer's Lactate on Non‐Lactate Metabolic Acidosis in Dogs

**DOI:** 10.1111/jvim.70099

**Published:** 2025-04-15

**Authors:** Roberto Rabozzi, Stefano Oricco

**Affiliations:** ^1^ Policlinico Veterinario Roma Sud, AniCura Roma Italy; ^2^ Centro Veterinario Imperiese Imperia Italy; ^3^ Department of Veterinary Sciences University of Parma Parma Italy

**Keywords:** acid–base, canine, electrolyte, fluid therapy, Stewart approach

## Abstract

**Background:**

Metabolic acidosis is a common acid–base disorder in critically ill dogs, with fluid therapy being a key but debated treatment. Sodium bicarbonate's risks have spurred interest in safer alternatives such as sodium lactate.

**Objectives:**

To compare the efficacy of a chloride‐free, high strong ion difference solution (H‐SID) to Ringer's lactate (RL) for treating metabolic acidosis, hypothesizing the superiority of the H‐SID solution.

**Animals:**

Forty‐six dogs with metabolic acidosis from two veterinary hospitals.

**Methods:**

Prospective randomized multicenter study. Dogs were randomly assigned to receive either RL or H‐SID at infusion rates of 4 or 10 mL/kg/h for 4 h, based on their volume status. H‐SID was compounded with sodium (145 mmol/L), lactate (145 mmol/L), potassium (10 mmol/L), and aspartate (10 mmol/L) in sterile water for injection.

**Results:**

The H‐SID group showed a significant increase in BE‐ecf (mmol/L) at infusion rates of 4 mL/kg/h (*p* < 0.001) and 10 mL/kg/h (*p* < 0.001) when compared to the RL group. At the lower infusion rate, the median increase was 4.1 mmol/L (95% CI: 3.37, 6.71), whereas the RL group exhibited a variation of −0.1 (95% CI: −0.75, 2.2). At the higher infusion rate, the median increase was 11 mmol/L (95% CI: 8.16, 12.52) compared to the RL group variation of 1.3 (95% CI: 0.01, 2.96).

**Conclusions and Clinical Importance:**

Our results indicate a significant alkalizing effect of the H‐SID solution in dogs with non‐lactic metabolic acidosis, demonstrating a superior effect compared to the RL solution without notable adverse effects.

**Abbreviations**
BE‐ecf: extracellular fluid base excess
Cl: chloride
Cl^−^ corr: corrected chloride
Hb: hemoglobin
HCO_3_
: bicarbonate
H‐SID: high strong ion difference
K^+^
: potassium
LVIDDn: left ventricular internal diameter in diastole normalized on body weight
Na^+^
: sodium
PCO_2_
: partial pressure of carbon dioxide
RL: Ringer's lactate
SID: strong ion difference
SIDa: apparent strong ion difference
SIDe: effective strong ion difference
SIG: strong ion gap

## 1 Introduction

Metabolic acidosis is one of the most frequent acid–base disorders in both human [1] and dog [2] critical care, and fluid therapy represents one of the most debated treatment options.

The effectiveness of treatments for metabolic acidosis using sodium bicarbonate has been re‐evaluated over the years, and confidence in its clinical efficacy remains low. There is conflicting evidence regarding its effectiveness, as seen in both randomized trials and systematic reviews of the literature. In 2021, the Sepsis Survival Campaign recommended against the use of sodium bicarbonate in human patients with septic shock and hypoperfusion‐induced lactic acidemia (weak recommendation, low quality of evidence) and suggested its use only in septic patients with pH ≤ 7.2 and AKI and AKIN score 2 or 3 (weak recommendation, low quality of evidence) [3].

The main criticisms of using sodium bicarbonate lie in the possibility of worsening intracellular acidosis even in the presence of alkalinization of the interstitial fluid [4] and the possibility of electrolyte imbalances associated with the rapid change in pH [5].

Bicarbonate is not the only alkalinizing anion that can be used in the clinical setting; sodium lactate is also an alkalinizing molecule with a high safety profile, several potential positive effects [6], and osmolar efficacy on dog red blood cells [7].

The primary aim of the study was to evaluate the efficacy of an iso‐osmolar, chloride‐free solution with a high strong ion difference (H‐SID), compared to a standard solution (Ringer's lactate [RL]) in hospitalized dogs with non‐lactate metabolic acidosis with or without renal failure.

The secondary objective was to describe the adverse effects, such as electrolyte imbalances, lactate accumulation, and any other side effects associated with the use of the two different solutions.

We hypothesized that an H‐SID solution would be more effective than RL in treating metabolic acidosis while minimizing treatment‐related adverse effects.

## 2 Materials and Methods

A prospective randomized multicenter study with Ethical Committee Autorisation number 233748 (University of PADUA—Italy) was designed to assess the efficacy and safety of two solutions in reversing metabolic acidosis in dogs and was performed at Policlinico Veterinario Roma Sud AniCura and Centro Veterinario Imperiese. The CONSORT guidelines were used to ensure the reporting of this clinical trial [8, 9, 10, 11].

Dogs privately owned and hospitalized for various diseases exhibiting different degrees of moderate or severe metabolic acidosis were included in the study. All of the venous blood examinations were performed using a blood gas analyzer (Stat Profile pHOx Ultra Blood Gas Analyzer Nova Biomedical, Waltham, MA, USA). Metabolic acidosis was defined as an extracellular fluid base excess (BE‐ecf) of −10 mmol/L or lower. At the time of inclusion, as previously published, dogs were classified as preload dependent or preload independent based on an echocardiographic index of fluid responsiveness [12]. The left ventricular internal diameter in diastole, normalized on body weight (LVIDDn) ≤ 1.34 cm/kg^0.294^ was used to classify dogs as hypovolemic. Normovolemic dogs were defined as having LVIDDn between 1.34 and 1.67 cm/kg^0.294^, while dogs with LVIDDn > 1.67 cm/kg^0.294^ were excluded due to a high probability of hypervolemia, indicating they do not require fluids. All dogs were assigned to the treatment groups through randomization using Random.org. They received either a solution with a low alkalinizing effect and low strong ion difference (SID), which was RL, or a solution with a high alkalinizing effect and H‐SID.

The chloride‐free isotonic solution with elevated strong ion difference (H‐SID) was prepared by adding 145 mmol/L of sodium lactate and 10 mmol/L of potassium aspartate to the sterile water for injections. The theoretical in vivo SID, depending on the sum of accompanying metabolizable anions, was equivalent to the sum of metabolizing accompanying anions and equal to 155 mmol/L. The solution used was considered iso‐osmolar based on the effects of sodium lactate as an effective osmole on the dog's red blood cells [7].

Normovolemic and hypovolemic dogs received 4 h of infusion at 4 or 10 mL/kg/h, respectively. Signalment, body weight, medical history, physical examination, underlying disease, and blood gas analysis, before fluid infusion (T0) and after 4 h of infusion (T4), were registered and compared for the following parameters: pH, PCO_2_, HCO_3_^−^ (mmol/L), Hb (g/dL), BE‐ecf (mmol/L), Na^+^ (mmol/L), K^+^ (mmol/L), Cl^−^ (mmol/L), Lactate (mmol/L), Cl^−^ corr (mmol/L), apparent SID (SIDa; mmol/L); other variables such as effective SID (SIDe; mmol/L), strong ion gap (SIG; mmol/L) were registered only before infusion (T0).

SIDa and SIDe, SIG, and corrected chloride were calculated using these formulas [13, 14, 15, 16, 17]:

$$\text{SIDa}={\mathrm{Na}}^{+}+K^{+}+{\mathrm{Ca}}^{2+}-\mathrm{Lac}-{\mathrm{Cl}}^{-}$$

$$\begin{array}{c} \text{SIDe}={{\mathrm{HCO}}_{3}}^{-}+\left(\mathrm{Alb}\times 10\times \left(0.123\times \mathrm{pH}\;0.631\right)\right)\\ \;+\left(\text{Phosphate}\times \left(0.309\times \mathrm{pH}\;0.469\right)\right)\\ \;\left[\mathrm{Alb}\;g/\mathrm{dL};\text{Phosphate mmol}/L\right]\;\\ \;\text{with Phosphate}\;\left(\text{mmol}/L\right)\\ \;=\mathrm{mg}/\mathrm{dL}\times 0.3229 \end{array}$$

SIG=SIDa−SIDe

$${\mathrm{Cl}}^{-}\;\text{corr}={\mathrm{Cl}}^{-}\times \left({{\mathrm{Na}}^{+}}_{\text{normal}}/{{\mathrm{Na}}^{+}}_{\mathrm{dog}}\right)$$

where Na^+^_normal_ was considered 146 mmol/L for dogs, according to our laboratory reference range and literature.

The theoretical framework suggested by Fencl and Leith [18], along with Kellum et al. [19], classifies the type of acidosis in the study population based on the primary influence of the total weak acid concentration (*A*_tot_), such as albumin or phosphorus, and the SID, which includes sodium, chloride, or lactate, on the observed BE‐ecf value. Any difference in the observed versus the expected sum of effects of SID or *A*_tot_ on BE‐ecf was justified by the presence of unknown ions and dog metabolic disturbance classified accordingly.

The exclusion criteria were as follows: (1) presence of lactic acidosis with a baseline lactate level greater than 4 mmol/L; (2) a pH value greater than 7.45; (3) presence of hypokalemia (K^+^ ≤ 3 mmol/L) that requires adjustment beyond the ranges provided by the solutions used in the study.

### 2.1 Statistical Analysis

Data distribution was assessed graphically and analytically. The normality of the variables' distribution was evaluated with the Shapiro–Wilk test, and nonparametric inferential tests were used according to the results. Descriptive statistics were used only for sex, age, body weight, breed, and underlying diseases.

The Wilcoxon test was used to evaluate differences within each group (RL and H‐SID) between baseline (T0) and post‐infusion (T4). The Mann–Whitney *U* test was employed to compare differences between the RL and H‐SID groups at baseline (T0) and after infusion (T4). Additionally, the Mann–Whitney *U* test was applied to assess differences in the changes (Δ) of specific variables between the RL and H‐SID groups.

Categorical variables between groups were evaluated using frequency, contingency tables, and Fisher's exact test.

Linear regression models were applied to the entire sample to explore associations between treatment effects, defined as changes in BE‐ecf, and independent variables, specifically changes in corrected Cl^−^ and SIDa.

The calculated sample size for the best chance to show a significant difference in medians within groups and between groups, assuming an effect size of 2 with a type I error of 0.05 and a power of 0.9, resulted in a minimum of six dogs and seven dogs per group, respectively.

Statistical significance was set to 5%.

Statistical analysis was performed using commercially available software (MedCalc Version 23.0.6, Ostend, Belgium; DATAtab e.U. Graz, Austria, and G*Power 3.1.9.6).

## 3 Results

A total of 46 hospitalized dogs from two veterinary hospitals were enrolled in the study between January and March 2018. These dogs exhibited metabolic acidosis, with BE‐ecf of −12.1 (IQR: −13.1, −10.7) mmol/L. Nineteen dogs were randomly assigned to receive the RL solution, while 27 dogs were allocated to receive the H‐SID solution. Five dogs enrolled in the study did not complete it due to various technical issues, resulting in data from 41 dogs being available for analysis (Figure S1). In the RL solution group, 16 dogs were analyzed: 9 were infused at a rate of 10 mL/kg/h (RL10 group) and 7 at a rate of 4 mL/kg/h (RL4 group). Additionally, in the H‐SID solution group, 25 dogs were analyzed: 13 were infused at a rate of 10 mL/kg/h (H‐SID10 group) and 12 at a rate of 4 mL/kg/h (H‐SID4 group).

The median age was 108 months (12–192), and the median weight was 13 kg (1.8–52), with 19 females and 22 males. There were no significant differences at T0 between groups in the demographic or baseline characteristics, including all acid–base parameters (T0, *p* > 0.05; Table 1).

**TABLE 1:** Demographic or baseline characteristics at the baseline in the population of dogs divided into four groups.

Variable	Groups								
	*N*	RL 4 mL/kg/h	*N*	RL 10 mL/kg/h	*N*	H‐SID 4 mL/kg/h	*N*	H‐SID 10 mL/kg/h	*p*
Sex (M/F)	7	5/2	9	4/5	12	4/8	13	6/7	0.457
Age (year)	7	10.0 (7.5–13.3)	9	7.0 (2.8–12.8)	12	8.5 (6.5–12.5)	13	10.0 (7.5–14.3)	0.500
Weight (kg)	7	16.0 (10.5–44.0)	9	13.0 (6.0–18.3)	12	13.5 (9.5–23.5)	13	11.0 (5.7–18.7)	0.471
pH	7	7.28 (7.24–7.30)	9	7.33 (7.25–7.35)	12	7.32 (7.30–7.37)	13	7.31 (7.26–7.35)	0.389
PCO_2_ (mmHg)	7	29.3 (28.3–34.5)	9	29.7 (24.0–32.4)	12	25.4 (23.8–28.1)	13	28.9 (24.1–30.5)	0.123
HCO_3_^−^ (mmol/L)	7	14.8 (14.1–15.8)	9	14.2 (13.5–15.4)	12	14.2 (13.3–15.1)	13	15.1 (13.0–16.3)	0.680
BE‐ecf (mmol/L)	7	−12.7 (−12.9 to −11.3)	9	−12.3 (−13.4 to −10.6)	12	−11.6 (−13.1 to −10.6)	13	−11.3 (−14.2 to −10.5)	0.982
Na^+^ (mmol/L)	7	143 (139–148)	9	149 (146–151)	12	145 (144–150)	13	147 (145–150)	0.154
K^+^ (mmol/L)	7	3.7 (3.4–5.4)	9	4.3 (3.5–5.4)	12	4.3 (3.8–4.6)	13	4.4 (3.9–5.1)	0.730
Cl^−^corr (mmol/L)	7	114 (110–118)	9	118 (110–119)	12	116 (116–118)	13	116 (113–118)	0.479
Ca^++^ (mmol/L)	7	1.38 (1.15–1.40)	9	1.31 (1.26–1.34)	12	1.38 (1.30–1.44)	13	1.31 (1.18–1.38)	0.251
SIDa (mmol/L)	7	34.9 (32.4–37.9)	9	32.9 (31.2–40.8)	12	33.4 (31.7–35.0)	13	34.0 (31.9–38.3)	0.663
SIDe (mmol/L)	7	26.8 (24.7–30.6)	9	25.9 (24.9–27.9)	12	25.4 (24.1–26.6)	13	26.2 (23.4–29.5)	0.683
SIG (mmol/L)	7	−8.1 (−8.9 to −6.4)	9	−7.6 (−10.7 to −6.6)	12	−7.8 (−10.6 to −6.8)	13	−8.9 (−9.6 to −7.9)	0.752
Lactate (mmol/L)	7	1.6 (0.5–2.2)	9	1.1 (1.0–2.1)	12	0.9 (0.6–1.5)	13	1.0 (0.9–1.8)	0.530
Hb (g/dL)	7	11.6 (10.3–12.5)	9	13.1 (8.7–15.0)	12	11.7 (10.7–13.1)	13	11.8 (9.5–14.6)	0.979

*Note:* Median and interquartile range (IQR) are reported for age, weight, and baseline acid–base and electrolyte values for each group: two solutions at two different infusion rates (Ringer's lactate 4 and 10 mL/kg/h and high‐SID 4 and 10 mL/kg/h). Significance between groups, assessed using the Kruskal–Wallis test, was set for *p* < 0.005.

Abbreviations: BE‐ecf: base excess extracellular fluid; Ca^++^: ionized calcium; Cl^−^corr: chloride corrected; Hb: hemoglobin; HCO_3_^−^: bicarbonate; K^+^: potassium; Lac: lactate; Na^+^: sodium; PCO_2_: partial pressure of carbon dioxide; SIDa: apparent strong ion difference; SIDe: effective strong ion difference; SIG: strong ion gap.

In the study population, we recorded 21/41 hyperchloremic acidosis, 7/41 acidosis due to the phosphorus effect, 6/41 due to unknown ions, and 7/41 with mixed effects. The distribution of the type of acidosis observed between groups was not different (*p* > 0.05).

### 3.1 Within‐Group Analysis (Comparison Before and After Infusion)

The 4 and 10 mL/kg/h infusion rates were examined separately. After 4 mL/kg/h RL infusion, a decrease in L^−^ (*p* = 0.042) and an increase in pH (*p* = 0.041) were observed. After 10 mL/kg/h RL infusion, there were increases in BE‐ecf (*p* = 0.024), HCO_3_^−^ (*p* = 0.015), and PCO_2_ (*p* = 0.020), along with a decrease in Hb (*p* = 0.012). After 4 mL/kg/h H‐SID infusion, increases in BE‐ecf (*p* = 0.002), HCO_3_^−^ (*p* = 0.002), L^−^ (*p* = 0.003), PCO_2_ (*p* = 0.021), pH (*p* = 0.018), SIDa (*p* = 0.002) were noted, with decreases in Cl^−^ (*p* = 0.003), corrected Cl^−^ (*p* < 0.001), and K^+^ (*p* = 0.045). After 10 mL/kg/h H‐SID infusion, increases were observed in BE‐ecf (*p* < 0.001), HCO_3_^−^ (*p* = 0.002), L^−^ (*p* = 0.003), PCO_2_ (*p* = 0.005), pH (*p* = 0.002), SIDa (*p* < 0.001), along with decreases in Cl^−^ (*p* < 0.001), corrected Cl^−^ (*p* < 0.001), and Hb (*p* = 0.006; Figures S2 and S3; Tables S1 and S2).

### 3.2 Between‐Group Comparison After Infusion

**FIGURE 1:**
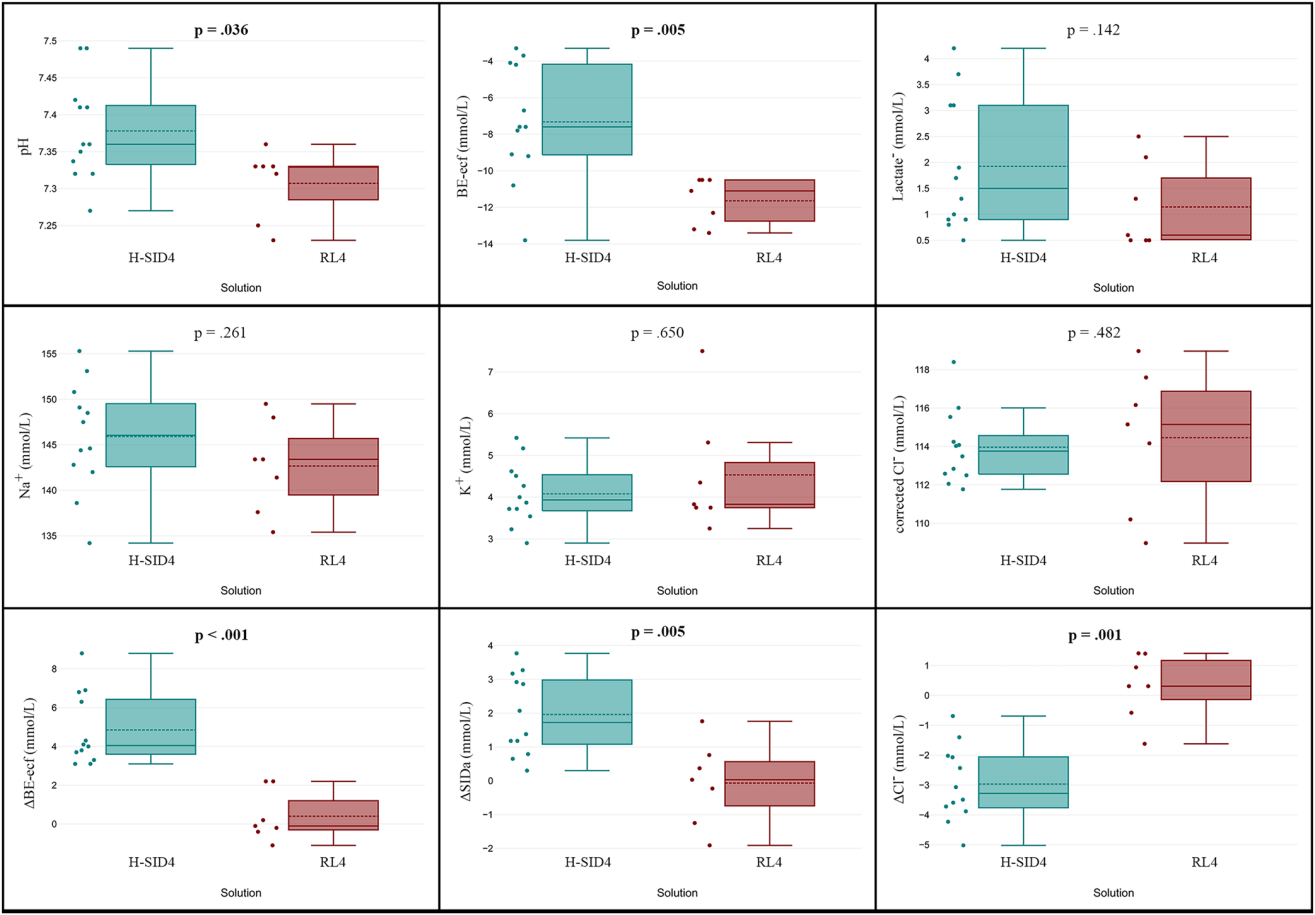
Box plots comparing post‐infusion values of pH, BE‐ecf, Lactate, Na^+^, K^+^, Cl^−^corr, ΔBE‐ecf, ΔSIDa, and ΔCl^−^ between two solutions (green: H‐SID; red: RL) after 4 h of infusion at a rate of 4 mL/kg/h. Differences between groups were analyzed, with *p*‐values displayed to indicate statistical significance (*p* < 0.005). BE‐ecf: base excess extracellular fluid; ΔBE‐ecf: change in BE; Cl^−^corr: corrected chloride; ΔCl^−^: change in Cl^−^; H‐SID: high‐SID; K^+^: potassium; Na^+^: sodium; RL: Ringer's lactate; ΔSIDa: change in apparent strong ion difference.

Following the 4 mL/kg/h infusion, statistically significant differences were found in median values between groups. Specifically, pH (*p* = 0.034), HCO_3_^−^ (*p* = 0.007), and BE‐ecf (*p* = 0.007) were higher in the H‐SID group compared to the RL group at T4 (Figure 1, Table S3).

**FIGURE 2:**
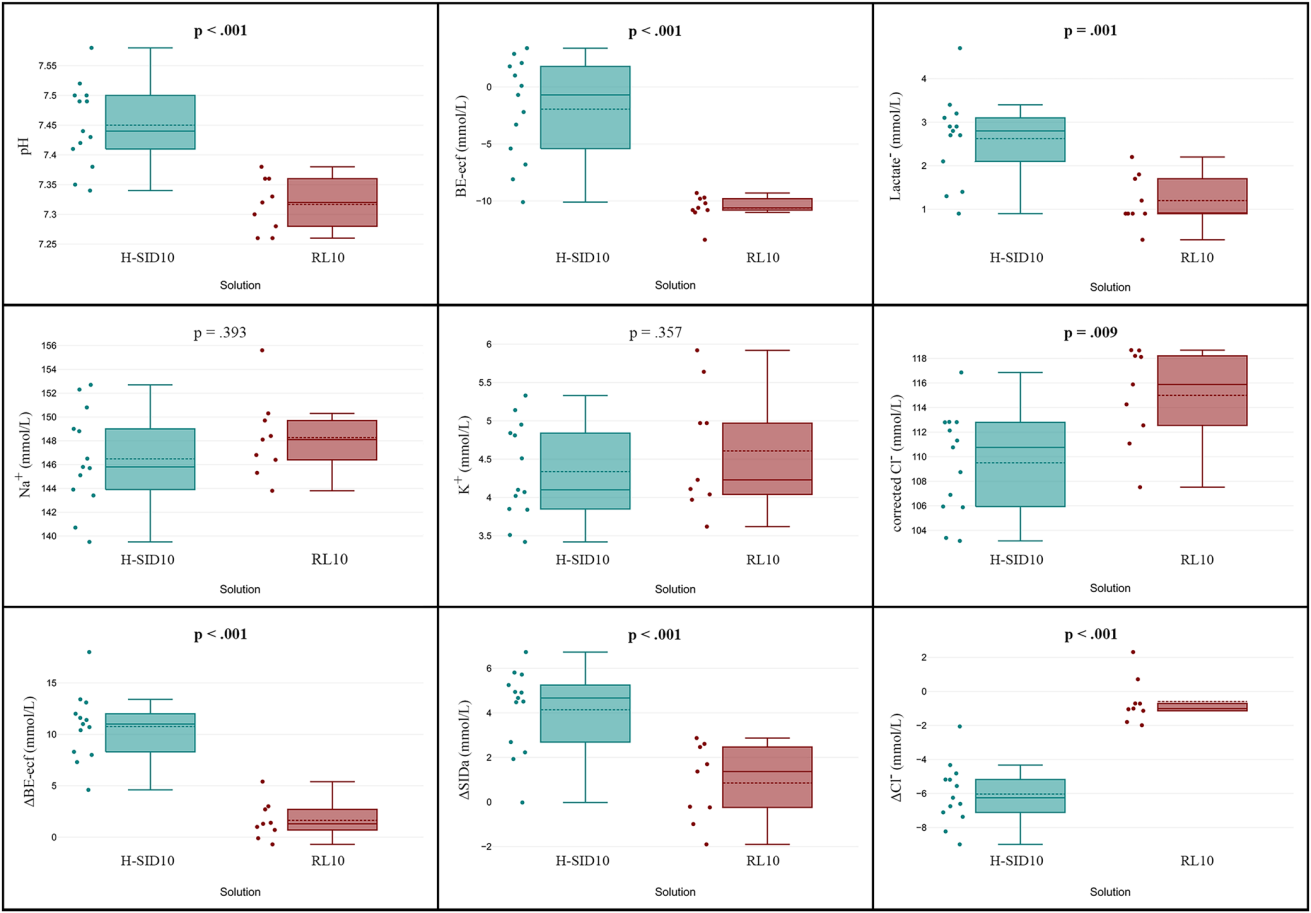
Box plots comparing post‐infusion values of pH, BE‐ecf, Lactate, Na^+^, K^+^, Cl^−^corr, ΔBE‐ecf, ΔSIDa, and ΔCl^−^ between two solutions (green: H‐SID; red: RL) after 4 h of infusion at a rate of 10 mL/kg/h. Differences between groups were analyzed, with *p*‐values displayed to indicate statistical significance (*p* < 0.005). BE‐ecf: base excess extracellular fluid; ΔBE‐ecf: change in BE; Cl^−^corr: corrected chloride; ΔCl^−^: change in Cl^−^; H‐SID: high‐SID; K^+^: potassium; Na^+^: sodium; RL: Ringer's lactate; ΔSIDa: change in apparent strong ion difference.

After the 10 mL/kg/h infusion, significant median differences between groups were observed. Specifically, the H‐SID group showed an increase in pH (*p* < 0.001), HCO_3_^−^ (*p* < 0.001), BE‐ecf (*p* < 0.001), and lactate (*p* = 0.002), and a decrease in Cl^−^ (*p* = 0.014) and corrected Cl^−^ (*p* = 0.009), compared to the RL group (Figure 2, Table S4).

The H‐SID group showed significant changes (Δ variation from T0 to T4) in BE‐ecf, Cl^−^, and SIDa compared to the RL group at both infusion rates (4 and 10 mL/kg/h; Tables 2 and 3, Figures 1 and 2).

**TABLE 2:** Differences in changes between groups at low‐rate infusion.

Variable	Ringer's lactate at 4 mL/kg/h			High‐SID at 4 mL/kg/h			*p*
	*N*	Median	95% CI	*N*	Median	95% CI	
ΔBE‐ecf (mmol/L)	7	−0.1	−0.75 to 2.2	12	4.1	3.37–6.71	< 0.001
ΔCl^−^ (mmol/L)	7	0.31	−1.11 to 1.41	12	−3.8	−3.85 to −2.03	< 0.001
ΔSIDa (mmol/L)	7	0.03	−1.58 to 1.26	12	1.73	0.86–3.13	0.005

*Note:* Median and 95% confidence interval (95% CI) are reported for the differences (Δ) between post‐infusion and baseline values for Ringer's lactate (RL) and High‐SID (H‐SID) at an infusion rate of 4 mL/kg/h. Statistical significance between groups was assessed using the Mann–Whitney *U* test, with significance set at *p* < 0.05.

Abbreviations: BE‐ecf: base excess extracellular fluid; Cl^−^: chloride; SIDa: apparent strong ion difference.

**TABLE 3:** Differences in changes between groups at high‐rate infusion.

Variable	Ringer's lactate at 10 mL/kg/h			High‐SID at 10 mL/kg/h			*p*
	*N*	Median	95% CI	*N*	Median	95% CI	
ΔBE‐ecf (mmol/L)	9	1.3	0.01–2.96	13	11.0	8.16–12.52	< 0.001
ΔCl^−^ (mmol/L)	9	−0.01	−1.71 to 0.51	13	−6.25	−7.23 to −5.01	< 0.001
ΔSIDa (mmol/L)	9	1.37	−0.89 to 2.59	13	4.67	2.48–5.47	0.001

*Note:* Median and 95% confidence interval (95% CI) are reported for the differences (Δ) between post‐infusion and baseline values for Ringer's lactate (RL) and high‐SID (H‐SID) at an infusion rate of 10 mL/kg/h. Statistical significance between groups was assessed using the Mann–Whitney *U* test, with significance set at *p* < 0.05.

Abbreviations: BE‐ecf: base excess extracellular fluid; Cl^−^: chloride; SIDa: apparent strong ion difference.

For BE‐ecf (mmol/L), at 4 mL/kg/h, the median increase in the H‐SID group was 4.1 mmol/L (95% CI: 3.37, 6.71), while the RL group showed a variation of −0.1 mmol/L (95% CI: −0.75, 2.2), *p* < 0.001. At 10 mL/kg/h, the median increase in the H‐SID group was 11 mmol/L (95% CI: 8.16, 12.52), compared to a change of 1.3 mmol/L (95% CI: 0.01, 2.96) in the RL group, *p* < 0.001. For Cl^−^ (mmol/L), at 4 mL/kg/h, the H‐SID group had a median change of −3.8 mmol/L (95% CI: −3.85, −2.03), while the RL group had a change of 0.31 mmol/L (95% CI: −1.11, 1.41), *p* < 0.001. At 10 mL/kg/h, the H‐SID group showed a median change of −6.25 mmol/L (95% CI: −7.23, −5.01), while the RL group's change was −0.01 mmol/L (95% CI: −1.71, 0.51), *p* < 0.001. For SIDa (mmol/L), at 4 mL/kg/h, the median increase in the H‐SID group was 1.73 mmol/L (95% CI: 0.86, 3.13), compared to a variation of −0.03 mmol/L (95% CI: −1.58, 1.26) in the RL group, *p* = 0.005. At 10 mL/kg/h, the H‐SID group had a median increase of 4.67 mmol/L (95% CI: 2.48, 5.47), while the RL group showed a change of 1.37 mmol/L (95% CI: −0.89, 2.59), *p* = 0.001.

### 3.3 Linear Regression Model

Linear regression models were constructed for all dogs to investigate the association between treatment effects (BE‐ecf changes) and independent variables (corrected Cl^−^ and SIDa changes).

The results of a linear fixed‐effects regression model showed that changes in SIDa were significantly correlated with changes in BE‐ecf, demonstrating a strong relationship (*R* ^2^ = 0.60; RSE = 3.02; *p* < 0.001). Moreover, changes in corrected chloride levels showed an even stronger correlation with changes in BE‐ecf (*R* ^2^ = 0.81; RSE = 2.09; *p* < 0.001).

### 3.4 Description of Adverse Effects

The following variations reflect increases in some dogs and decreases in others, depending on individual responses to the infusions.

After a 4 mL/kg/h RL infusion, sodium levels showed a median change of −0.8 mmol/L (IQR: −0.9, 0.6). Potassium levels changed by 0.1 mmol/L (IQR: −0.3, 0.1). Calcium levels changed by 0.01 mmol/L (IQR: −0.03, 0.02). Lactate levels changed by a median of −0.2 mmol/L (IQR: −0.3, −0.03).

After a 10 mL/kg/h RL infusion, sodium levels showed a median change of 0.1 mmol/L (IQR: −0.6, 1.2). Potassium levels changed by 0.1 mmol/L (IQR: −0.7, 0.6). Calcium levels changed by 0.01 mmol/L (IQR: −0.003, 0.003). Lactate levels changed by a median of −0.2 mmol/L (IQR: −1.0, 0.1).

After a 4 mL/kg/h H‐SID infusion, sodium levels showed a median change of 0.0 mmol/L (IQR: −0.9, 0.9). Potassium levels changed by −0.3 mmol/L (IQR: −0.4, 0.1). Calcium levels changed by −0.03 mmol/L (IQR: −0.07, −0.02). Lactate levels changed by a median of 0.5 mmol/L (IQR: 0.2, 0.6).

After a 10 mL/kg/h H‐SID infusion, sodium levels showed a median change of −1.0 mmol/L (IQR: −1.7, −0.4). Potassium levels changed by −0.3 mmol/L (IQR: −0.7, −0.01). Calcium levels changed by −0.1 mmol/L (−0.14, −0.04). Lactate levels changed by a median of 1.1 mmol/L (0.5, 2.0).

Throughout the H‐SID infusion, neither lactate nor pH exceeded the predefined thresholds of 5 mmol/L and 7.5, respectively, and no clinical signs associated with the infusion of either solution were observed.

## 4 Discussion

Compared to RL solution, this study shows a clear advantage of the H‐SID solution in treating metabolic acidosis in hospitalized dogs.

Metabolic acidosis is a common concern in critically ill patients, affecting 6%–40% of individuals depending on diagnostic criteria and patient demographics [1, 20]. It is also frequently observed in critically ill dogs and cats [2]. This condition often arises from a combination of underlying causes, resulting in “mixed acid–base disorders.” Factors such as hypoalbuminemia or SID alkalosis can obscure its presentation, complicating diagnosis and management. Importantly, it is associated with increased mortality in intensive care settings [21]. Critical indicators such as low pH, diminished base excess, and elevated lactic acid levels are independent predictors of mortality [22]. These findings emphasize the importance of accurate monitoring and effective management of acid–base imbalances to improve clinical outcomes. To effectively address this challenge, a systematic approach incorporating physiochemical methods, such as the Stewart method [23], enables accurate identification of the underlying causes of metabolic acidosis, particularly in complex or mixed cases. This clarity provides a solid foundation for guiding appropriate treatment and ultimately enhancing dog care. The treatment of metabolic acidosis should be directed at the underlying condition, not only the acidosis per se. Fluid therapy with alkalinizing solutions should not be considered the first‐line treatment and in cases of metabolic acidosis resulting from reversible factors, such as in individuals who are hypoperfused or hypoxemic, using high SID solutions can lead to the risk of excessive alkalinization by the end of the treatment.

The therapeutic approaches to metabolic acidosis are currently a topic of significant debate [24]. Balanced solutions are the standard of care in both human and veterinary medicine for managing fluid therapy in patients with metabolic acidosis; however, sodium bicarbonate is also commonly described, especially in veterinary medicine. However, there is no clear evidence coming from recent meta‐analyses or systematic reviews about any positive effect of sodium bicarbonate therapy on patient outcomes, and the systematic review published by Fujii et al. [25] gives no evidence to support the use of sodium bicarbonate in metabolic acidosis. Furthermore, the use of sodium bicarbonate to correct severe acidemia may be tempting to clinicians, but previous studies also have shown some important side effects. First, intracellular acidosis, due to a rapid increase in carbon dioxide concentration; bicarbonate reacts with acids to form water and CO_2_, which, unlike charged ions such as HCO_3_^−^ and H^+^, rapidly diffuses into the intracellular compartment and rehydrates, driving the same reaction in reverse (from water and CO_2_ to HCO_3_^−^ and H^+^), with a rapid decrease in intracellular pH. Second, bicarbonate may negatively affect cardiac contractility and, if administered as a hypertonic solution, it may depress vasomotor tone with peripheral vasodilation [26, 27]. Third, electrolyte imbalances such as hypocalcemia, hypernatremia, and hypokalemia are quite often present in patients treated with sodium bicarbonate, with arrhythmogenicity and hyperosmolarity [28].

One alternative to sodium bicarbonate is sodium lactate. Various positive effects of lactate infusion have been observed in vivo. Lactate has been shown to serve as an energy source for myocardial and neuronal metabolism, as demonstrated in previous studies [29, 30]. It does not exhibit negative inotropic effects during infusion [31] and functions as an effective osmole for canine red blood cells [7] and the blood–brain barrier [32]. Moreover, lactate infusion has positively affected microcirculation during endotoxic shock in a porcine model [33, 34]. Additional benefits include its potential to reduce renal microvascular thrombosis associated with disseminated intravascular coagulation [34], alongside its possible protective role against endothelial dysfunction [31]. Beyond these systemic effects, lactate infusion has demonstrated specific benefits in traumatic brain injury by reversing brain oxygenation and metabolism dysfunction through vasodilatory, mitochondrial, and anti‐edema mechanisms [35, 36]. These multifaceted effects position lactate as a promising therapeutic option for the management of critically ill patients [6]. Moreover, sodium lactate infusion has the advantage over sodium bicarbonate as it causes a very slow rise in the PCO_2_ levels and probably less intracellular acidosis.

The alkalinizing or acidifying effect of infusion solutions has been known since 1999 when Scheingraber et al. described a hyperchloremic acidosis after a rapid saline infusion in a group of women, afterward confirmed by other authors with mathematical models, and in vitro and in vivo experimental studies [37, 38, 39, 40]. Stewart's theory better explains the rationale for changing pH during crystalloid infusion using a quantitative approach, which is clearer than a qualitative approach based on bicarbonate. The Stewart approach [23, 41] is a physiochemical, quantitative tool for acid–base interpretation. All the variables involved in the acid–base balance are clearly highlighted, and all the interactions and relationships between these factors are well‐defined. Any variation in PCO_2_, SID, *A*_tot_ leads to a variation in pH, except for a proportional variation of all the acid–base determinants; in vitro experiments have shown that a dilution of the plasma in a closed system generates a null effect on the pH, regardless of the type of solution and the fraction of dilution applied; however, if the same experiment is carried out in an open system which maintains the PCO_2_ at physiological values, without being influenced by the dilution, the effect on the pH is manifested [39]. The dilutional acidosis observed during saline infusion, especially during high dilution fraction, is due to the rehydration of the carbon dioxide, which creates carbonic acid. Every infusion solution is electrically balanced and has a zero SID immediately after administration. However, solutions that contain no electrolytes, such as 5% glucose, or only non‐metabolizable ions, like 0.9% sodium chloride (NaCl), will maintain a SID of 0. In contrast, solutions such as RL contain lactate and other metabolized ions measured in millimoles per liter. These ions are metabolized after infusion and affect the dog's pH differently. Imagine a theoretical scenario in which 1 L of plasma is infused with a solution of known SID, under conditions of infinite dilution. In this scenario, the concentration of the diluent greatly exceeds that of the plasma, causing the plasma to adopt the characteristics of the diluent. Consequently, the total weak acid concentration (*A*_tot_) becomes negligible, while the SID remains identical to that of the infusion solution. Under the assumption of an open system with a normal PCO_2_, the bicarbonate concentration [HCO_3_^−^] fills the SID. Based on these assumptions, an infused solution with SID higher than the dog's baseline [HCO_3_^−^] values will have an alkalizing effect, and a solution with SID lower than the dog's baseline [HCO_3_^−^] values will have an acidifying effect. Assuming the same SID of the solution, the effect's magnitude is influenced by the dilution applied, with a more significant effect occurring with increased volume infused, as during volume replacement, and minor effects during fluid maintenance. A solution with a SID equal to the dog's baseline [HCO_3_^−^] values will not affect pH, regardless of the fractional dilution applied.

The H‐SID solution was composed of 145 mmol/L of sodium, 145 mmol/L of lactate, 10 mmol/L of potassium, and 10 mmol/L of aspartate; therefore, the calculated SID of this solution was equivalent to the sum of metabolizing accompanying anions (145 mmol/L of lactate and 10 mmol/L of aspartate), equal to 155 mmol/L; for this reason, this free chloride solution has an alkalizing effect ever, regardless of the dog's bicarbonate levels. The H‐SID solution at both infusion rates demonstrated a significant effect on the changing pH, PCO_2_, HCO_3_^−^, BE‐ecf, SIDa, Ca^2+^, and L^−^ after treatment, compared to the RL solution, with a clinically significant effect on pH, HCO_3_^−^, BE‐ecf, and SIDa. The alkalizing effect of the H‐SID infusion was associated with a significant decrease in chloride levels, resulting in an increased SID. Following infusion at 4 mL/kg/h, between‐group comparisons of absolute chloride and SIDa values did not show statistically significant differences. However, the within‐group analysis demonstrated significant changes in chloride and SIDa from T0 to T4 in the H‐SID group, with no notable changes in the RL group. The lack of significant differences in absolute post‐infusion values may be attributed to high variance in chloride and SIDa levels within the H‐SID group and the limited sample size (seven dogs) for the H‐SID solution at this infusion rate. Nonetheless, the strongly significant delta variation in both variables highlights the distinct effects of the two solutions, even at the lower infusion rate. The observed alkalizing effect linked to chloride variation was evident in the strong relationship between changes in chloride and SIDa and even more pronounced in the regression between changes in chloride and base excess. The significant effect observed on chloride levels after infusion is not considered a side effect but rather a desired outcome aimed at increasing the SID. Reducing chloride levels through the use of low‐chloride or chloride‐free solutions mimics a compensatory mechanism observed in patients with metabolic acidosis, helping to minimize the risk of chloride‐associated adverse effects [42, 43, 44, 45, 46, 47]. However, the clinical significance and evidence supporting the potential benefits of moderate chloride reduction remain to be fully elucidated.

The sodium concentration remained unchanged during the infusion, regardless of the type of solution used. The compound solution was iso‐osmolar, having a sodium level similar to that of plasma. This similarity likely explains the lack of effect observed, contrasting with previous reports in humans following the infusion of hypertonic sodium lactate solutions [48]. In contrast, administering a hypertonic solution, such as half‐molar sodium lactate, resulted in hypernatremia and hypokalemia, consistent with findings from earlier studies [49, 50]. The rise in natremia is attributable to the sodium load delivered, which markedly increases plasma osmolality. Sodium serves as the osmotic backbone of the extracellular compartment, making it the primary determinant of plasma osmolality.

The potassium concentration remained unchanged during the RL solution but decreased with the H‐SID solution. Specifically, the results showed statistically significant differences at low‐rate infusions, whereas they were not significant at high‐rate infusions, even though the trend was consistent in both cases. This result is particularly interesting given that the electrolyte concentration in the solution (10 mmol/L) is higher than the plasmatic level. Literature shows that potassium concentration is influenced by hydrogen ion concentration, which in turn affects pH levels. During acidosis, potassium shifts from the intracellular space to the extracellular space, increasing its presence in the plasma. In contrast, during alkalosis, potassium moves in the opposite direction, shifting back into the cells. The mechanism is often simplified as an exchange between potassium ions and hydrogen ions. When acidosis occurs, the level of hydrogen ions increases, prompting potassium ions to exit the cell. Conversely, during alkalosis, hydrogen ion levels decrease, causing potassium ions to enter the cell. The fluctuations influence this dynamic exchange in plasma levels of these ions. However, due to the significantly different plasma concentrations, with hydrogen ions having a normal concentration of 40 nmol/L and potassium at 4 mmol/L, this cannot be simply explained by direct exchange. During acidosis, there is an inhibition of the Na^+^–H^+^ exchange and Na^+^–HCO_3_^−^ cotransport, which reduces the entry of sodium. This, in turn, decreases the effectiveness of the Na^+^,K^+^‐ATPase pump, leading to an increase in the plasma concentration of potassium. Additionally, stimulation of the Cl^−^–HCO_3_^−^ exchange raises the intracellular concentration of chloride and activates the K^+^–Cl^−^ cotransport, resulting in a shift of potassium outside the cell. The opposite effect occurs in cases of metabolic alkalosis [51]. During the infusion of a solution with a high SID, the alkalinizing effect causes potassium to shift into the cells. This shift counteracts the intake of potassium from the solution and can even lead to a net loss of this electrolyte. This phenomenon is particularly noticeable during inorganic metabolic disturbances such as hyperchloremic acidosis or hypochloremic alkalosis. It is less evident during other metabolic disturbances and is absent in respiratory disturbances [51].

Another expected result was a decrease in hemoglobin levels after the infusion at a higher rate. However, given the significant results observed for both solutions, it is evident that the change in hemoglobin value was influenced by the infusion rate rather than the type of solution used.

Lastly, the movement of PCO_2_ during infusion shows a significant increase both during the infusion of H‐SID and at a high rate of RL infusion. This indicates a counterbalance between ventilation and changes in metabolic acid–base balance. In the case of metabolic acidosis, an increase in ventilation results in a decrease in PCO_2_, which helps mitigate the impact on pH. This is evidenced by the coexistence of low base excess and relatively normal pH levels. Similarly, during alkalinization, PCO_2_ increases while the effect on pH is reduced.

The lactate levels significantly increased after the infusion of the H‐SID and were directly related to the rate applied; however, the effect was not clinically significant, with a median value of 2.8 mmol/L and a maximum value of 4.7 mmol/L after infusion at a high rate (10 mL/kg/h).

The results suggest that the solution is likely safe; however, the primary focus of this study was its efficacy rather than safety. While no adverse effects were observed in our population of 25 dogs treated with H‐SID, we cannot draw definitive conclusions about the solution's safety. Furthermore, the possibility of more pronounced effects in dogs with different co‐morbidities cannot be ruled out, and the solution's safety should be evaluated in future studies with a larger sample size. In any case, the clinical impact of a rapid, iatrogenic increase in lactate levels due to infusion in a patient with normal perfusion or baseline lactate levels that are normal or only slightly elevated has not been proven to be comparable to an increase resulting from hypoperfusion [52]. It is important to note that this is not necessarily the case. A potential limitation of using lactate‐rich solutions is the diminished reliability of lactate as a predictor of mortality in critically ill patients [53].

Post hoc power analysis (two‐tailed test) conducted as a follow‐up analysis demonstrated excellent results in our population. Based on our findings, with a sample size of 40 dogs and *α* = 0.05, the calculated effect size was 2.27, yielding an estimated power of 0.996. The effect was well powered for this study; however, these results do not allow us to conclude that the H‐SID solution is entirely free of side effects in this population. While we can assert that side effects such as electrolyte imbalances are unlikely to occur more frequently than 1 in 20 cases, we cannot entirely exclude the possibility of their occurrence.

The population of this study is well consistent with the theoretically best efficacy of this type of high SID solution; indeed, respiratory acidosis or lactic acidosis would not be equally responsive. The resolution of hypoventilation or hypoperfusion is mandatory for treating these types of acidosis, and using an alkalizing solution could be counter‐indicated, as it may cause an excessive increase in pH once the underlying cause of the acid–base disturbance has resolved. The clinical target of this type of solution is not the volume replacement but the qualitative treatment of electrolyte and acid–base imbalance, acting on the patient's chloride levels and, consequently, on the patient's SID value [38, 54].

This study has several limitations. Firstly, the absence of albumin and phosphate measurements after the infusion prevents us from calculating the SIDe and the buffer base. Secondly, all dogs were enrolled based on the BE‐ecf value, including those with a pH greater than 7.2. Thirdly, the cut‐off value for BE‐ecf was selected arbitrarily and is not supported by existing literature, as there is no recognized cut‐off to classify the severity of the disorder. Lastly, due to the sample size and the number of dogs, we could not perform any subgroup analysis based on the type of acidosis when the cohort was divided accordingly.

In conclusion, this study emphasizes the significant effects of the H‐SID solution in dogs experiencing moderate to severe non‐lactic metabolic acidosis, while noting the absence of any clinically relevant adverse effects. These findings suggest that this solution could be a promising option for treating critically ill dogs in the years to come.

## Disclosure

Authors declare no off‐label use of antimicrobials.

## Ethics Statement

Authors declare no Institutional Animal Care and Use Committee or other approval was needed due to the retrospective nature of this study. Authors declares human ethics approval was not needed.

## Conflicts of Interest

The authors declare no conflicts of interest.

## Supporting information


**Figure S1.** Participant flow diagram.

**Figure S2.** Paired median and interquartile range plots illustrating pre‐ and post‐infusion values for pH, BE‐ecf, K^+^, L^−^, Cl^−^corr, and SIDa following the administration of two different solutions at a rate of 4 mL/kg/h. Connecting lines between pre‐ and post‐infusion points highlight individual changes. Statistical significance was set at *p* < 0.005. BE‐ecf: base excess extracellular fluid; Cl^−^corr: corrected chloride; H‐SID: high‐SID; K^+^: potassium; L^−^: lactate; RL: Ringer’s lactate; SIDa: apparent strong ion difference.

**Figure S3.** Paired median and interquartile range plots illustrating pre‐ and post‐infusion values for pH, BE‐ecf, K^+^, L^−^, Cl^−^corr, and SIDa following the administration of two different solutions at a rate of 10 mL/kg/h. Connecting lines between pre‐ and post‐infusion points highlight individual changes. Statistical significance was set at *p* < 0.005. BE‐ecf: base excess extracellular fluid; Cl^−^corr: corrected chloride; H‐SID: high‐SID; K^+^: potassium; L^−^: lactate; RL: Ringer’s lactate; SIDa: apparent strong ion difference.

**Table S1.** Supporting information.

**Table S2.** Supporting information.

**Table S3.** Supporting information.

**Table S4.** Supporting information.
