# Supplementary material for: Effects of an Iso‐Osmotic Chloride‐Free Solution With High Strong Ion Difference vs. Ringer's Lactate on Non‐Lactate Metabolic Acidosis in Dogs
Source: J Vet Intern Med. 2025 Apr 15;39(3):e70099. doi: 10.1111/jvim.70099 (PMC12000541; doi:10.1111/jvim.70099)
Supplement: Supplementary file 1 — Figure S1. Participant flow diagram. [file JVIM-39-e70099-s006.doc]

**CONSORT 2010 Flow Diagram**

**Allocation**

**Enrollment**

**Follow-Up**

**Analysis**

Assessed for eligibility (n= 46)

Excluded (n= 0 )

  Not meeting inclusion criteria (n= )

  Declined to participate (n= )

  Other reasons (n= )

Lost to follow-up (missing information or technical issues) (n= 2)

Discontinued intervention (n= 0)

Ringer's Lactate solution (RL) (n= 19)

 Received allocated intervention (n= 19)

 Did not receive allocated intervention (n= 0)

High-strong ion difference solution (H-SID) (n= 27)

 Received allocated intervention (n=27)

 Did not receive allocated intervention) (n=0)

Randomized (n= 46)

Low infusion rate

(4 mL/Kg/h) (n= 9)

High infusion rate

(10 mL/Kg/h) (n= 10)

Low infusion rate

(4 mL/Kg/h) (n= 12)

High infusion rate

(10 mL/Kg/h) (n= 15)

Lost to follow-up (missing information or technical issues) (n= 1)

Discontinued intervention (n= 0)

Lost to follow-up (missing information or technical issues) (n= 0)

Discontinued intervention (n= 0)

Lost to follow-up (missing information or technical issues) (n= 2)

Discontinued intervention (n= 0)

Analysed (n=7)

Excluded from analysis (n= 0)

Analysed (n=9)

Excluded from analysis (n= 0)

Analysed (n=12)

Excluded from analysis (n= 0)

Analysed (n=13)

Excluded from analysis (n= 0)
