# Supplementary material for: Effects of an Iso‐Osmotic Chloride‐Free Solution With High Strong Ion Difference vs. Ringer's Lactate on Non‐Lactate Metabolic Acidosis in Dogs
Source: J Vet Intern Med. 2025 Apr 15;39(3):e70099. doi: 10.1111/jvim.70099 (PMC12000541; doi:10.1111/jvim.70099)
Supplement: Supplementary file 4 — Table S1. Supporting information. [file JVIM-39-e70099-s003.docx]

| Variable | **Ringer’s Lactate 4 ml/kg/h** | | | | | **H-SID 4 ml/kg/h** | | | | |
| --- | --- | --- | --- | --- | --- | --- | --- | --- | --- | --- |
|  | N | RL T0 | RL T4 | Differences | *P* value | N | H-SID T0 | H-SID T4 | Differences | *P* value |
| pH | 7 | 7.28 (7.24 - 7.30) | 7.33 (7.27 - 7.33) | 0.04 (0.01 - 0.05) | **.041** | 12 | 7.32 (7.30 - 7.37) | 7.36 (7.33 - 7.41) | 0.05 (0.04 - 0.08) | **.018** |
| PCO_2_ (mmHg) | 7 | 29.3 (28.3 - 34.5) | 29.3 (28.8 - 30.8) | -1.9 (-4.2 - 0.0) | .080 | 12 | 25.4 (23.8 – 28.1) | 29.9 (26.0 - 32.5) | 2.3 (1.4 - 5.4) | **.021** |
| HCO_3_^-^ (mmol/L) | 7 | 14.8 (14.1 - 15.8) | 15.1 (14.6 - 15.6) | -0.4 (-0.7 - 1.4) | .672 | 12 | 14.2 (13.3 – 15.1) | 17.5 (16.7 - 20.1) | 3.7 (2.7 - 5.1) | **.002** |
| BE-ecf (mmol/L) | 7 | -12.7 (-12.9 - -11.3) | -11.1 (-13.0 - -10.5) | -0.1 (-0.4 - 1.7) | .799 | 12 | -11.6 (-13.1 - -10.6) | -7.6 (-9.2 - -4.2) | 4.1 (3.5 - 6.6) | **.002** |
| Na^+^ (mmol/L) | 7 | 143 (139 - 148) | 143 (139 - 147) | -0.8 (-0.9 - 0.6) | .175 | 12 | 145 (144 - 150) | 146 (142 - 150) | 0.0 (-0.9 - 0.9) | .969 |
| K^+^ (mmol/L) | 7 | 3.7 (3.4 - 5.4) | 3.8 (3.8 - 5.1) | 0.1 (-0.3 - 0.1) | .866 | 12 | 4.3 (3.8 - 4.6) | 3.9 (3.6 - 4.6) | -0.3 (-0.4 - 0.1) | **.045** |
| Cl^-^ (mmol/L) | 7 | 114 (106 - 117) | 113 (105 - 117) | 0.0 (-0.6 - 0.6) | .916 | 12 | 119 (114 - 121) | 114 (111 - 117) | -3.3 (-3.6 - -2.5) | **.003** |
| Cl^-^corr (mmol/L) | 7 | 114 (110 - 118) | 115 (111 - 117) | 0.3 (-0.4 - 1.3) | .578 | 12 | 116 (116 - 118) | 114 (113 - 115) | -3.3 (-3.8 - -2.1) | **< .001** |
| Ca^++^ (mmol/L) | 7 | 1.38 (1.15 - 1.40) | 1.35 (1.17 - 1.35) | 0.01 (-0.03 - 0.02) | .865 | 12 | 1.38 (1.30 - 1.44) | 1.34 (1.27 - 1.41) | -0.03 (-0.07 - -0.02) | **.041** |
| SIDa (mmol/L) | 7 | 34.9 (32.4 - 37.9) | 34.7 (32.8 - 38.2) | -0.03 (-1.0 - 0.66) | 1.000 | 12 | 33.4 (31.7 - 35.0) | 35.0 (34.4 - 37.6) | 1.73 (1.0 - 3.1) | **.002** |
| Lactate (mmol/L) | 7 | 1.6 ( .5 - 2.2) | 0.6 (0.5 - 1.9) | -0.2 (-0.3 - -0.03) | **.042** | 12 | .9 ( .6 - 1.5) | 1.5 (0.9 - 3.1) | 0.5 (0.2 - 0.6) | **.003** |
| Hb (g/dL) | 7 | 11.6 (10.3 - 12.5) | 10.9 (10.0 - 12.2) | -2.3 (-10.1 - -1.3) | .498 | 12 | 11.7 (10.7 - 13.1) | 11.3 (10.4 - 12.6) | -0.2 (-0.8 - 0.2) | .209 |

**Table S1**. Changes in acid-base and electrolytes after treatment with Ringer’s lactate or H-SID solutions at low-rate infusion

Median and interquartile range (IQR) are presented for baseline (T0), post-infusion (T4), and changes in acid-base and electrolyte values for Ringer's lactate and High-SID administered at an infusion rate of 4 mL/kg/h. Variables include: BE-ecf: base excess extracellular fluid; Ca^++^: ionized calcium; Cl^-^: chloride; Cl^-^corr: chloride corrected; Hb: hemoglobin; HCO_3_^-^: bicarbonate; K^+^: potassium; Lac: lactate; Na^+^: sodium; PCO_2_: partial pressure of carbon dioxide; SIDa: apparent strong ion difference. Statistical significance between groups was assessed using the Wilcoxon test, with significance set at *P* < .05.
