# Supplementary material for: Effects of an Iso‐Osmotic Chloride‐Free Solution With High Strong Ion Difference vs. Ringer's Lactate on Non‐Lactate Metabolic Acidosis in Dogs
Source: J Vet Intern Med. 2025 Apr 15;39(3):e70099. doi: 10.1111/jvim.70099 (PMC12000541; doi:10.1111/jvim.70099)
Supplement: Supplementary file 5 — Table S2. Supporting information. [file JVIM-39-e70099-s004.docx]

**Table S2.** Changes in acid-base and electrolytes after treatment with Ringer’s lactate or H-SID solutions at high-rate infusion

| Variable | **Ringer’s Lactate 10 ml/kg/h** | | | | | **H-SID 10 ml/kg/h** | | | | |
| --- | --- | --- | --- | --- | --- | --- | --- | --- | --- | --- |
|  | N | RL T0 | RL T4 | Differences | *P* value | N | H-SID T0 | H-SID T4 | Differences | *P* value |
| pH | 9 | 7.33 (7.25 - 7.35) | 7.32 (7.28 - 7.36) | -0.01 (-0.01 - 0.04) | .719 | 13 | 7.31 (7.26 - 7.35) | 7.44 (7.40 - 7.50) | 0.14 (0.10 - 0.17) | **.002** |
| PCO_2_ (mmHg) | 9 | 29.7 (24.0 - 32.4) | 33.5 (26.9 - 35.3) | 1.8 (1.2 - 5.0) | **.021** | 13 | 28.9 (24.1 - 30.5) | 30.0 (27.4 - 36.7) | 4.0 (0.9 - 6.4) | **.005** |
| HCO_3_^-^ (mmol/L) | 9 | 14.2 (13.5 - 15.4) | 16.4 (16.1 - 16.4) | 1.1 (0.6 - 2.3) | **.015** | 13 | 15.1 (13.0 – 16.3) | 23.5 (19.4 - 26.3) | 8.7 (6.4 - 9.2) | **.002** |
| BE-ecf (mmol/L) | 9 | -12.3 (-13.4 - -10.6) | -10.6 (-10.9 - -9.8) | 1.3 (0.5 - 2.8) | **.024** | 13 | -11.3 (-14.2 - -10.5) | -0.7 (-5.8 - 1.9) | 11.0 (8.2 - 12.3) | **< .001** |
| Na^+^ (mmol/L) | 9 | 149 (146 - 151) | 148 (146 - 150) | 0.1 (-0.6 - 1.2) | .674 | 13 | 147 (145 - 150) | 146 (144 - 149) | -1.0 (-1.7 - -0.4) | **.008** |
| K^+^ (mmol/L) | 9 | 4.3 (3.5 - 5.4) | 4.2 (4.0 - 5.1) | 0.1 (-0.7 - 0.6) | .734 | 13 | 4.4 (3.9 - 5.1) | 4.1 (3.9 - 4.9) | -0.3 (-0.7 - -0.01) | .064 |
| Cl^-^ (mmol/L) | 9 | 120 (114 - 122) | 119 (113 - 121) | 0.0 (-1.1 - 0.8) | .624 | 13 | 120 (111 - 122) | 112 (104 - 114) | -7.0 (-8.6 - -5.2) | **< .001** |
| Cl^-^corr (mmol/L) | 9 | 118 (110 - 119) | 116 (112 - 118) | -1.0 (-1.3 - -0.4) | .138 | 13 | 116 (113 - 118) | 111 (106 – 113) | -6.3 (-7.2 - -5.1) | **< .001** |
| Ca^++^ (mmol/L) | 9 | 1.31 (1.26 - 1.34) | 1.33 (1.23 - 1.37) | 0.01 (-0.003 - 0.03) | .205 | 13 | 1.31 (1.18 - 1.38) | 1.21 (1.13 - 1.28) | -0.10 (-0.14 - -0.04) | **.017** |
| SIDa (mmol/L) | 9 | 32.9 (31.2 - 40.8) | 35.8 (32.5 - 39.7) | 1.4 (-0.4 - 2.5) | .250 | 13 | 34.0 (31.9 - 38.3) | 38.2 (36.5 - 43.1) | 4.7 (2.6 - 5.4) | **< .001** |
| Lactate (mmol/L) | 9 | 1.1 (1.0 - 2.1) | 0.9 (0.9 - 1.7) | -0.2 (-1.0 - 0.1) | .213 | 13 | 1.0 ( .9 - 1.8) | 2.8 (1.9 - 3.1) | 1.1 (0.5 - 2.0) | **.003** |
| Hb (g/dL) | 9 | 13.1 (8.7 - 15.0) | 11.8 (7.6 - 14.1) | -0.9 (-1.3 - -0.7) | **.013** | 13 | 11.8 (9.5 - 14.6) | 11.2 (8.9 - 14.0) | -1.0 (-1.5 - -0.3) | **.006** |

Median and interquartile range (IQR) are presented for baseline (T0), post-infusion (T4), and changes in acid-base and electrolyte values for Ringer's lactate and High-SID administered at an infusion rate of 10 mL/kg/h. Variables include: BE-ecf: base excess extracellular fluid; Ca^++^: ionized calcium; Cl^-^: chloride; Cl^-^corr: chloride corrected; Hb: hemoglobin; HCO_3_^-^: bicarbonate; K^+^: potassium; Lac: lactate; Na^+^: sodium; PCO_2_: partial pressure of carbon dioxide; SIDa: apparent strong ion difference. Statistical significance between groups was assessed using the Wilcoxon test, with significance set at *P* < .005.
