# Supplementary material for: Effects of an Iso‐Osmotic Chloride‐Free Solution With High Strong Ion Difference vs. Ringer's Lactate on Non‐Lactate Metabolic Acidosis in Dogs
Source: J Vet Intern Med. 2025 Apr 15;39(3):e70099. doi: 10.1111/jvim.70099 (PMC12000541; doi:10.1111/jvim.70099)
Supplement: Supplementary file 6 — Table S3. Supporting information. [file JVIM-39-e70099-s002.docx]

**Table S3**. Changes in acid-base and electrolytes after treatment with Ringer’s lactate or H-SID solutions at low-rate infusion

| Variable | **Ringer’s Lactate 4 ml/kg/h** | | **H-SID 4 ml/kg/h** | |  |
| --- | --- | --- | --- | --- | --- |
|  | N | RL T4 | N | H-SID T4 | *P* value |
| pH | 7 | 7.33 (7.27 - 7.33) | 12 | 7.36 (7.33 - 7.41) | **.034** |
| PCO_2_ (mmHg) | 7 | 29.3 (28.8 - 30.8) | 12 | 29.9 (26.0 - 32.5) | .866 |
| HCO_3_^-^ (mmol/L) | 7 | 15.1 (14.6 - 15.6) | 12 | 17.5 (16.7 - 20.1) | **.010** |
| BE-ecf (mmol/L) | 7 | -11.1 (-13.0 - -10.5) | 12 | -7.6 (-9.2 - -4.2) | **.007** |
| Na^+^ (mmol/L) | 7 | 143 (139 - 147) | 12 | 146 (142 - 150) | .237 |
| K^+^ (mmol/L) | 7 | 3.8 (3.8 - 5.1) | 12 | 3.9 (3.6 - 4.6) | .612 |
| Cl^-^ (mmol/L) | 7 | 113 (105 - 117) | 12 | 114 (111 - 117) | .837 |
| Cl^-^corr (mmol/L) | 7 | 115 (111 - 117) | 12 | 114 (113 - 115) | .482 |
| Ca^++^ (mmol/L) | 7 | 1.35 (1.17 - 1.35) | 12 | 1.34 (1.27 - 1.41) | .865 |
| SIDa (mmol/L) | 7 | 34.7 (32.8 - 38.2) | 12 | 35.0 (34.4 - 37.6) | .902 |
| Lactate (mmol/L) | 7 | 0.6 (0.5 - 1.9) | 12 | 1.5 (0.9 - 3.1) | .126 |
| Hb (g/dL) | 7 | 10.9 (10.0 - 12.2) | 12 | 11.3 (10.4 - 12.6) | .584 |

Median and interquartile range (IQR) are reported post-infusion (T4) in acid-base and electrolyte values for Ringer's lactate and High-SID at an infusion rate of 4 mL/kg/h. Variables include: BE-ecf: base excess extracellular fluid; Ca^++^: ionized calcium; Cl^-^: chloride; Cl^-^corr: chloride corrected; Hb: hemoglobin; HCO_3_^-^: bicarbonate; K^+^: potassium; Lac: lactate; Na^+^: sodium; PCO_2_: partial pressure of carbon dioxide; SIDa: apparent strong ion difference. Statistical significance between groups was assessed using the Mann-Whitney U test, with significance set at *P* < .005.
