# Supplementary material for: Effects of an Iso‐Osmotic Chloride‐Free Solution With High Strong Ion Difference vs. Ringer's Lactate on Non‐Lactate Metabolic Acidosis in Dogs
Source: J Vet Intern Med. 2025 Apr 15;39(3):e70099. doi: 10.1111/jvim.70099 (PMC12000541; doi:10.1111/jvim.70099)
Supplement: Supplementary file 7 — Table S4. Supporting information. [file JVIM-39-e70099-s005.docx]

**Table S4**. Changes in acid-base and electrolytes after treatment with Ringer’s lactate or H-SID solutions at high-rate infusion

| Variable | **Ringer’s Lactate 10 ml/kg/h** | | **H-SID 10 ml/kg/h** | |  |
| --- | --- | --- | --- | --- | --- |
|  | N | RL T4 | N | H-SID T4 | *P* value |
| pH | 9 | 7.32 (7.28 - 7.36) | 13 | 7.44 (7.40 - 7.50) | **< .001** |
| PCO_2_ (mmHg) | 9 | 33.5 (26.9 - 35.3) | 13 | 30.0 (27.4 - 36.7) | .896 |
| HCO_3_^-^ (mmol/L) | 9 | 16.4 (16.1 - 16.4) | 13 | 23.5 (19.4 - 26.3) | **< .001** |
| BE-ecf (mmol/L) | 9 | -10.6 (-10.9 - -9.8) | 13 | -0.7 (-5.8 - 1.9) | **< .001** |
| Na^+^ (mmol/L) | 9 | 148 (146 - 150) | 13 | 146 (144 - 149) | .393 |
| K^+^ (mmol/L) | 9 | 4.2 (4.0 - 5.1) | 13 | 4.1 (3.9 - 4.9) | .333 |
| Cl^-^ (mmol/L) | 9 | 119 (113 - 121) | 13 | 112 (104 - 114) | **.014** |
| Cl^-^corr (mmol/L) | 9 | 116 (112 - 118) | 13 | 111 (106 – 113) | **.009** |
| Ca^++^ (mmol/L) | 9 | 1.33 (1.23 - 1.37) | 13 | 1.21 (1.13 - 1.28) | .053 |
| SIDa (mmol/L) | 9 | 35.8 (32.5 - 39.7) | 13 | 38.2 (36.5 - 43.1) | .096 |
| Lactate (mmol/L) | 9 | 0.9 (0.9 - 1.7) | 13 | 2.8 (1.9 - 3.1) | **.002** |
| Hb (g/dL) | 9 | 11.8 (7.6 - 14.1) | 13 | 11.2 (8.9 - 14.0) | .920 |

Median and interquartile range (IQR) are reported post-infusion (T4) in acid-base and electrolyte values for Ringer's lactate and High-SID at an infusion rate of 10 mL/kg/h. Variables include: BE-ecf: base excess extracellular fluid; Ca^++^: ionized calcium; Cl^-^: chloride; Cl^-^corr: chloride corrected; Hb: hemoglobin; HCO_3_^-^: bicarbonate; K^+^: potassium; Lac: lactate; Na^+^: sodium; PCO_2_: partial pressure of carbon dioxide; SIDa: apparent strong ion difference. Statistical significance between groups was assessed using the Mann-Whitney U test, with significance set at *P* < .005.
